# Characteristics of Women Enrolled into a Randomized Clinical Trial of Dapivirine Vaginal Ring for HIV-1 Prevention

**DOI:** 10.1371/journal.pone.0128857

**Published:** 2015-06-10

**Authors:** Thesla Palanee-Phillips, Katie Schwartz, Elizabeth R. Brown, Vaneshree Govender, Nyaradzo Mgodi, Flavia Matovu Kiweewa, Gonasagrie Nair, Felix Mhlanga, Samantha Siva, Linda-Gail Bekker, Nitesha Jeenarain, Zakir Gaffoor, Francis Martinson, Bonus Makanani, Sarita Naidoo, Arendevi Pather, Jessica Phillip, Marla J. Husnik, Ariane van der Straten, Lydia Soto-Torres, Jared Baeten

**Affiliations:** 1 Wits Reproductive Health and HIV Research Institute, University of the Witswatersrand, School of Clinical Medicine, Johannesburg, South Africa; 2 FHI 360, Durham, NC, United States of America; 3 Statistical Center for HIV/AIDS Research and Prevention, Fred Hutchinson Cancer Research Center, Seattle, WA, United States of America; 4 HIV Prevention Research Unit, South African Medical Research Council, Durban, South Africa; 5 University of Zimbabwe—University of California San Francisco Collaborative Research Program (UZ-UCSF) Research Collaboration, Harare, Zimbabwe; 6 Makerere University-Johns Hopkins University Research Collaboration, Kampala, Uganda; 7 Centre for AIDS Programme of Research in SA (CAPRISA), Durban, South Africa; 8 Desmond Tutu HIV Foundation Clinical Research Site, Cape Town, South Africa; 9 UNC Lilongwe Clinical Research Site, Lilongwe, Malawi; 10 College of Medicine-John Hopkins University Research Project Queen Elizabeth Central Hospital, Blantyre, Malawi; 11 RTI International, San Francisco, CA, United States of America; 12 Division of AIDS, National Institute of Allergy and Infectious Diseases, National Institutes of Mental Health, and Eunice Shriver Kennedy, National Institute of Child Health and Human Development, National Institutes of Health, Bethesda, MD, United States of America; 13 Departments of Global Health, Medicine, and Epidemiology, University of Washington, Seattle, WA, United States of America; University of Toronto, CANADA

## Abstract

**Introduction:**

Women in sub-Saharan Africa are a priority population for evaluation of new biomedical HIV-1 prevention strategies. Antiretroviral pre-exposure prophylaxis is a promising prevention approach; however, clinical trials among young women using daily or coitally-dependent products have found low adherence. Antiretroviral-containing vaginal microbicide rings, which release medication over a month or longer, may reduce these adherence challenges.

**Methods:**

ASPIRE (A Study to Prevent Infection with a Ring for Extended Use) is a phase III, randomized, double-blind, placebo-controlled trial testing the safety and effectiveness of a vaginal ring containing the non-nucleoside reverse transcriptase inhibitor dapivirine for prevention of HIV-1 infection. We describe the baseline characteristics of African women enrolled in the ASPIRE trial.

**Results:**

Between August 2012 and June 2014, 5516 women were screened and 2629 HIV-1 seronegative women between 18–45 years of age were enrolled from 15 research sites in Malawi, South Africa, Uganda, and Zimbabwe. The median age was 26 years (IQR 22–31) and the majority (59%) were unmarried. Nearly 100% of participants reported having a primary sex partner in the prior three months but 43% did not know the HIV-1 status of their primary partner; 17% reported additional concurrent partners. Nearly two-thirds (64%) reported having disclosed to primary partners about planned vaginal ring use in the trial. Sexually transmitted infections were prevalent: 12% had *Chlamydia trachomatis*, 7% *Trichomonas vaginalis*, 4% *Neisseria gonorrhoeae*, and 1% syphilis.

**Conclusions:**

African HIV-1 seronegative women at risk of HIV -1 infection were successfully enrolled into a phase III trial of dapivirine vaginal ring for HIV-1 prevention.

## Introduction

Over half of the 24.7 million people living with HIV-1 infection in sub-Saharan Africa in 2013 were women [1]. Unprotected heterosexual intercourse is the leading mode of HIV-1 transmission amongst women in this region. While a number of HIV-1 prevention interventions have demonstrated varying effectiveness in reducing HIV-1 risk, including behavior change, use of male and female condoms, treatment of sexually transmitted infections (STIs), male circumcision, knowledge of HIV-1 status, and uptake of antiretroviral treatment to reduce the infectiousness of persons with HIV-1, HIV-1 rates in young African women in many settings remain unacceptably high. This is, in part, because many HIV-1 prevention methods require the participation or consent of a male partner and therefore current prevention options may prove inadequate for many women, who may be unable to negotiate HIV-1 or STI testing and treatment or condom use with their partners [2]. Developing woman-controlled HIV-1 prevention options that women can use remains therefore a global priority.

Microbicides are products being developed for topical application inside the vagina or rectum with the intention of reducing the sexual acquisition of HIV-1 and/or other STIs. Vaginal microbicides offer the promise of a tool to prevent HIV-1 that would be under a woman’s control and could complement other HIV-1 prevention strategies. Microbicides containing highly specific HIV-1 antiretroviral medications are one form of HIV-1 pre-exposure prophylaxis (PrEP), in which a HIV-1 susceptible individual uses oral or topical antiretroviral medications for prevention of HIV-1 acquisition [3]. Randomized trials of the antiretroviral agent tenofovir, formulated as oral pills or a topical vaginal gel, demonstrated efficacy in diverse at-risk populations worldwide [4–8]. However, in two trials among young women at risk for HIV-1 (VOICE and FEM-PrEP), adherence to daily tenofovir-containing pills or vaginal gels was very low and HIV-1 prevention efficacy could not be assessed [9–11]. As a result, development of potent, antiretroviral-based prevention products not requiring daily or coitally-dependent adherence has become a priority.

Vaginal rings have been developed and marketed as delivery methods for medications, capable of providing sustained and controlled release of active ingredients such as hormonal contraception and estrogen replacement therapy. In recent years, there has been considerable interest in developing similar ring devices for the administration of microbicidal compounds to prevent vaginal acquisition of HIV-1 and other STIs [12]. Intended to be worn continuously, coitally-independent microbicide rings would in theory maintain effective vaginal microbicide concentrations over many weeks or months, thereby addressing challenges around timing of product application and adherence associated with other prophylaxis strategies. Dapivirine, a substituted di-amino-pyrimidine derivative with potent activity against HIV-1 as a non-nucleoside reverse-transcriptase inhibitor (NNRTI), has been formulated into a silicone elastomer vaginal microbicide ring. Preclinical studies and multiple early-stage clinical trials have evaluated the safety of dapivirine in oral, gel, and vaginal ring formulations [13–18]. These clinical trials support a favourable safety profile and tolerability of dapivirine in general and specifically in vaginal delivery form.

The Microbicide Trials Network (MTN)-020 trial, ASPIRE (A Study to Prevent Infection with a Ring for Extended Use) is a phase III, randomized, double-blind, placebo-controlled trial evaluating the safety and effectiveness of dapivirine vaginal ring inserted once every four weeks for the prevention of HIV-1 infection in healthy, HIV-1 uninfected sexually active women. Here, we describe the design of the trial and baseline characteristics of the ASPIRE cohort.

## Methods

### Ethics statement

Prior to implementation, the study protocol was reviewed and approved by ethics review committees at collaborating institutions at each of the study sites (National Health Sciences Research Committee of Malawi and Johns Hopkins University Bloomberg School of Public Health Institutional Review Board [Malawi: Blantyre site], National Health Sciences Research Committee of Malawi and University of North Carolina at Chapel Hill Institutional Review Board [Malawi: Lilongwe site], University of Cape Town: Human Research Ethics Committee [South Africa: Cape Town site], Biomedical Research Ethics Administration, University of KwaZulu-Natal [South Africa: Durban—eThikwini site], South African Medical Research Council Ethics Committee [South Africa: Durban—Botha's Hill, Chatsworth, Isipingo, Tongaat, Umkomaas, Verulam sites], Wits Human Research Ethics Committee, University of Witwatersrand [South Africa: Johannesburg site], Joint Clinical Research Centre Institutional Review Board and Johns Hopkins University School of Medicine Institutional Review Board [Uganda: Kampala site], Medical Research Council of Zimbabwe and Committee on Human Research, University of California—San Francisco [Zimbabwe: Chitungwiza-Seke South, Chitungwiza-Zengeza, and Harare-Spilhaus sites]) and the Prevention Sciences Review Committee of the National Institute of Allergy and Infectious Diseases of the US National Institutes of Health. All participants provided written informed consent.

### Study design

ASPIRE is a phase III, multi-site, randomized, double-blind, parallel-arm, placebo-controlled trial of the dapivirine vaginal ring for the prevention of HIV-1 acquisition in healthy sexually active HIV-1 uninfected women (ClinicalTrials.gov number NCT01617096). The primary study objectives are to determine the effectiveness and safety of the dapivirine vaginal ring when compared against the placebo ring when inserted once every four weeks over the investigational product use period. Secondary objectives include assessment of the acceptability of and adherence to the dapivirine vaginal ring, the frequency of antiretroviral drug resistance in women who acquire HIV-1 during the study period, and the relationship between drug concentrations and HIV-1 seroconversion. The study protocol can be found at http://www.mtnstopshiv.org/studies/3614.

The trial was designed with 90% power to detect a minimum 60% reduction in risk of HIV-1 infection for the primary effectiveness objective, using a log-rank test having a one-sided false positive error rate of 0.025. A minimum lower bound for the effectiveness of the study product was set at 25%, consistent with the design of other trials of novel HIV-1 prevention interventions and with a goal of establishing that a novel product has clear public health benefit[19–21]. Under these assumptions, a minimum of 120 HIV-1 acquisitions events was estimated to be required. Because an endpoint-driven design was used, the trial will continue until the target number of HIV-1 endpoints is accrued. Assuming a background HIV-1 incidence of 3.9% per year (i.e., in the placebo arm), based on HIV-1 incidence in a prior microbicide trial in a similar population [21] and allowing for a loss to follow-up rate of 1% per month, an initial sample size of 3476 women was planned. After the trial had initiated, another microbicide trial, conducted at the same or similar study sites, demonstrated a higher than anticipated background HIV-1 incidence, at over 5% per year [22]. The trial sample size for ASPIRE was then recalculated to approximately 2600 subjects.

### Study procedures

The study is being conducted at fifteen research sites in four African countries: Malawi (Blantyre and Lilongwe), South Africa (Cape Town, Durban [7 sites], Johannesburg), Uganda (Kampala), and Zimbabwe (Chitungwiza [2 sites], Harare). Participants were recruited from a variety of sources across sites, including clinics for treatment of STIs, family planning clinics, post-natal clinics, as well as community-based locations. Advice regarding recruitment materials was sought from site community representatives before use.

The trial’s inclusion and exclusion criteria are detailed in Table 1. Enrolled women were assigned in a 1:1 ratio to one of the two study arms: to receive either a silicone elastomer vaginal matrix ring containing 25 mg of dapivirine or a placebo vaginal ring. At this enrollment visit, women are provided with vaginal ring use instructions by clinical staff and counselled on what to do in cases of ring expulsion. They then demonstrate the ability to remove and reinsert the ring. Finally, correct ring placement is assessed by digital exam by clinical staff. The study product is manufactured by the International Partnership for Microbicides (Silver Spring, MD). A fixed block size of 12, stratified by site, was used to generate randomization assignments.

**Table 1:** Study Inclusion and Exclusion Criteria.

**Inclusion criteria**	Women must meet all of the following criteria to be eligible for inclusion in the study.
	**1)** Age 18 through 45 years (inclusive) at screening,
	**2)** Able and willing to provide written informed consent to be screened for and to take part in the study
	**3)** Able and willing to provide adequate locator information
	**4)** HIV uninfected based on testing performed at screening and enrollment
	**5)** Per participant report, sexually active, defined as having vaginal intercourse at least once in the 3 months prior to screening
	**6)** Using an effective method of contraception at enrollment, and intending to use an effective method for the duration of study participation; effective methods include hormonal methods (except contraceptive rings); intrauterine device (IUD); and sterilization (of participant)
	**7)** At screening and enrollment, agrees not to participate in other research studies involving drugs, medical devices, vaginal products, or vaccines for the duration of study participation
	*Note*: *Tampons may be used for the duration of the trial*.
**Exclusion criteria**	Women who meet any of the following criteria will be excluded from the study.
	**1)** Per participant report at screening:
	a. Intends to become pregnant during study participation
	b. Plans to relocate away from the study site during study participation
	c. Plans to travel away from the study site for more than 8 consecutive weeks during study participation
	**2)** Pregnant
	**3)** Currently breastfeeding
	**4)** Diagnosed with urinary tract infection (UTI), unless treated and symptoms are resolved prior to enrollment
	**5)** Diagnosed with pelvic inflammatory disease, an STI or reproductive tract infection requiring treatment per current WHO guidelines, unless treated and symptoms are resolved prior to enrollment
	**6)** Has a clinically apparent Grade 2 or higher pelvic exam finding. Cervical bleeding associated with speculum insertion and/or specimen collection judged to be within the range of normal is not exclusionary.
	**7)** Participant report and/or clinical evidence of any of the following:
	a. Known adverse reaction to any of the study products (ever)
	b. Known adverse reaction to latex (ever)
	c. Chronic vaginal candidiasis
	d. Non-therapeutic injection drug use in the 12 months prior to Screening
	e. Post-exposure prophylaxis (PEP) for HIV-1 exposure within 6 months prior to enrollment
	f. Last pregnancy outcome 90 days or less prior to enrollment
	g. Gynaecologic or genital procedure (e.g., tubal ligation, dilation and curettage, piercing) 90 days or less prior to enrollment
	h. Participation in any other research study involving drugs, medical devices, vaginal products, or vaccines, within 60 days of enrollment
	i. Participation in any HIV prevention study using systemic or topical antiretroviral medications, within 12 months of enrollment
	j. As determined by the site investigator, any significant uncontrolled active or chronic cardiovascular, renal, liver, hematologic, neurologic, gastrointestinal, psychiatric, endocrine, respiratory, immunologic disorder or infectious disease, including active tuberculosis
	**8)** Has any of the following laboratory abnormalities at Screening Visit:
	a. Aspartate aminotransferase (AST) or alanine transaminase (ALT) Grade 1 or higher
	b. Creatinine Grade 2 or higher
	c. Hemoglobin Grade 2 or higher
	d. Platelet count Grade 1 or higher
	e. Pap result Grade 2 or higher
	**9)** Has any other condition that, in the opinion of the site investigator, would preclude informed consent, make study participation unsafe, complicate interpretation of study outcome data, or otherwise interfere with achieving the study objectives

*Note*: *Grading of clinical and laboratory abnormalities is defined* per the National Institute of Allergy and Infectious Diseases Division of AIDS Table for Grading the Severity of Adult and Pediatric Adverse Events Version 1.0, December, 2004 (Clarification dated August 2009) and the Female Genital Grading Table for Use in Microbicide Studies Addendum 1 to the Division of AIDS Table for Grading Adult and Pediatric Adverse Events, Version 1.0, December 2004 (Clarification dated August 2009)

Participants are followed monthly, with HIV-1 counseling and serologic testing, pregnancy testing, safety monitoring, and product provision. HIV-1 testing is by two HIV-1 rapid tests (Oraquick, Unigold, or Determine), conducted in parallel and performed at screening, enrollment, and monthly follow-up. During follow-up, a positive result for either (or both) rapid tests triggers temporary product hold and further evaluation for confirmation of HIV-1 infection by HIV-1 Western blot and RNA PCR testing. Women with confirmed HIV-1 acquisition are permanently discontinued from the study product but continue with follow-up; all HIV-1 seroconverters were referred to local services for HIV-1 care and support. Women who become pregnant are temporarily discontinued from the study product for the duration of pregnancy and breastfeeding but remain in follow-up. Referrals for pregnancy and HIV-1 care are provided. Participants will be followed for four weeks after last study product use visit to measure the potential for delayed HIV-1 seroconversion. Individualized adherence counseling is conducted at each monthly visit. Returned used rings and quarterly plasma samples are archived for testing for the presence of dapivirine, as objective markers of adherence.

All participants receive a package of HIV-1 prevention services at each study visit, including HIV-1 and STI risk reduction counselling and free condoms. Etiologic testing and treatment for STIs for participants and partner (where possible) was done at screening and during follow-up, including for syphilis (rapid plasma reagin [RPR] screening test followed by a confirmatory microhemagglutinin assay for *Treponema pallidum* [MHA-TP] or *Treponema pallidum* haemagglutination assay [TPHA] for reactive samples), *Neisseria gonorrhoeae and Chlamydia trachomatis* (the Becton Dickenson Probe Tec strand displacement assay, and *Trichomonas vaginalis* (OSOM Rapid Trichomonas test); treatment was provided on site. Contraception was provided free-of-charge at each research site, and all sites were encouraged to offer a broad mix of methods.

Site staff entered demographic, behavioural, and clinical data related to screening and enrollment onto standard paper-based case report forms. Each form was faxed using DataFax software (DF/Net Software ULC) and received at the Statistical and Data Management Center located in Seattle, Washington, USA. Two independent data technicians verified the data on each form. Audio computer assisted self-interview (ACASI) was also used for behavioural data collection. ACASI data were transmitted electronically and securely to the trial’s data management center. Data analyses were conducted using SAS version 9.2 (SAS Institute, Cary, NC).

## Results

Between August 2012 and June 2014, 5516 women were screened for study eligibility and 2629 were enrolled, with a screen-to-enroll ratio of 2.1:1. Fifty-four percent of those enrolled were from South Africa, with 10% each from Malawi and Uganda, and 26% from Zimbabwe (Table 2). Women’s ages spanned the full extent of the eligibility age range, from 18 to 45 years, with a median age of 26 years (interquartile range [IQR] 22–31), 39% <25 years of age and 14% ≥35 years of age. Most participants from the South African sites were unmarried (92%) compared to Uganda (34%), Malawi (15%), and Zimbabwe (17%), and 85% of all enrolled participants completed some secondary schooling or higher.

**Table 2:** Participant characteristics^1^.

	Malawi	South Africa	Uganda	Zimbabwe	All Countries
**Participants enrolled, N**	272	1426	253	678	2629
**Age, years (median, IQR)**	28 (24, 33)	24 (21, 29)	28 (24, 33)	28 (25, 33)	26 (22, 31)
**Currently unmarried**	41 (15%)	1313 (92%)	87 (34%)	112 (17%)	1553 (59%)
**Highest level of education**					
No schooling	9 (3%)	3 (0.2%)	9 (4%)	2 (0.3%)	23 (1%)
Primary school (partial and complete)	140 (51%)	46 (3%)	117 (46%)	78(12%)	381 (14%)
Secondary school (partial and complete)	121 (44%)	1245 (87%)	112 (44%)	592 (87%)	2070 (79%)
Attended college or university	2 (1%)	132 (9%)	15 (6%)	6 (1%)	155 (6%)
**Had a primary sex partner during the past 3 months**	271 (100%)	1420 (100%)	252 (100%)	673 (99%)	2616 (100%)
**Number of other sex partners in the past 3 months**					
None	251 (92%)	1219 (85%)	135 (53%)	585 (86%)	2190 (83%)
1	12 (4%)	180 (13%)	72 (28%)	40 (6%)	304 (12%)
2 or more	9 (3%)	27 (2%)	46 (18%)	53 (8%)	135 (5%)
**Number of vaginal sex acts in the past 3 months (median, IQR)**	36 (16, 48)	10 (5, 20)	12 (6, 30)	48 (36, 70)	20 (7, 36)
**Type of condom used in the last act of vaginal sex**					
Male condom	103 (38%)	944 (66%)	80 (32%)	347 (51%)	1474 (56%)
Female condom	1 (0.4%)	14 (1%)	2 (1%)	10 (1%)	27 (1%)
Neither	167 (61%)	463 (32%)	169 (67%)	321 (47%)	1120 (43%)
**Anal sex in the past 3 months**	2 (1%)	43 (3%)	5 (2%)	4 (1%)	54 (2%)
**In past year, received money, material goods, gifts, drugs or shelter in exchange for vaginal or anal sex**	16 (6%)	88 (6%)	38 (15%)	21 (3%)	163 (6%)
**Primary partner knows of participation in the trial**	259 (95%)	1008 (71%)	76 (30%)	629 (93%)	1972 (75%)
**Primary partner knows has been asked to use ring**	249 (92%)	807 (57%)	48 (19%)	576 (86%)	1680 (64%)
**Primary partner HIV status**					
HIV positive	4 (1%)	16 (1%)	2 (1%)	13 (2%)	35 (1%)
HIV negative	196 (72%)	928 (65%)	113 (45%)	207 (31%)	1444 (55%)
Participant does not know	71 (26%)	476 (34%)	137 (54%)	453 (67%)	1137 (43%)
**Had same primary partner for last 3 months**	262 (97%)	1399 (98%)	220 (87%)	657 (98%)	2538 (97%)
**Current method of contraception**					
Intrauterine device	6 (2%)	79 (6%)	71 (28%)	169 (25%)	325 (12%)
Oral contraceptives	6 (2%)	242 (17%)	14 (6%)	18 (3%)	280 (11%)
Injectable contraceptives	115 (42%)	1046 (73%)	112 (44%)	179 (26%)	1452 (55%)
Depot medroxyprogesterone acetate (DMPA)	115 (100%)	666 (64%)	112 (100%)	179 (100%)	1072 (74%)
Norethisterone enanthate (NET-EN)	0 (0%)	380 (36%)	0 (0%)	0 (0%)	380 (26%)
Hormonal implant	120 (44%)	21 (1%)	49 (19%)	311 (46%)	501 (19%)
Sterilization	25 (9%)	42 (3%)	7 (3%)	3 (0.4%)	77 (3%)
**STIs detected at the screening visit**					
*Trichomonas vaginalis*	28 (10%)	88 (6%)	13 (5%)	51 (8%)	180 (7%)
*Neisseria gonorrhoeae*	13 (5%)	55 (4%)	15 (6%)	26 (4%)	109 (4%)
*Chlamydia trachomatis*	6 (2%)	237 (17%)	25 (10%)	48 (7%)	316 (12%)
*Syphilis* ^*2*^	11 (4%)	7 (0.5%)	6 (2%)	15 (2%)	39 (1%)

^1^Note that the denominators used to calculate percentages throughout this table may vary owing to small amounts of missing data for individual variables and may not sum to 100% due to rounding.

^2^ Based on a reactive result to the syphilis screening and a positive confirmatory test result. Note that participants testing positive at screening were provided treatment according to WHO guidelines.

Nearly 100% of participants reported having a primary sex partner during the 3 months prior to trial enrollment, and 17% reported additional partners in this period. The median number of sex acts during the 3 months prior to trial enrollment was 20 (IQR 7–36), and 57% reported male or female condom use with the last vaginal sex act. Anal sex in these prior three months was reported by 2% of those enrolled (n = 54), and transactional sex in the past year was reported by 6%

Out of 2616 participants reporting a primary sex partner in the past 3 months, 1.3% (n = 35) knew their primary partner was HIV-1 infected while 43% did not know their primary partner’s HIV-1 serostatus. Seventy-five percent of participants reported that their primary partner was aware of their participation in the research study, but this level of disclosure varied across the countries from 95% in Malawi to 30% in Uganda. Sixty-four percent reported that their primary partner knew that they would be using a vaginal ring, from 92% in Malawi to 19% in Uganda

Baseline contraceptive use reflects a wide method mix: 55% of women reported use of injectable contraception with the majority of those (74%) using injectable depot medroxyprogesterone acetate and 26% injectable norethisterone enanthate, 11% used combined oral contraceptives, 12% intrauterine devices, and 19% contraceptive implants (Table 2).

The most common STI detected at the screening visit was *C*. *trachomatis*, found in 12% of the cohort and with the highest prevalence reported at the South African sites (17%). This was followed by *T*. *vaginalis* in 7%, *N*. *gonorrhoeae* in 4%, and syphilis in 1% of participants.

Of 5516 women screened, 2887 (52%) did not enroll (Table 3), of whom 2454 completed screening but were not eligible and 378 did not complete screening. Only 55 women (1% of those screened) were eligible but declined enrollment. Among the 2454 ineligible women, the most common reason was seropositivity for HIV-1 (35%, 854 women). Other reasons included pregnancy or planning to become pregnant (8%, 203 women), breastfeeding (1%, 31 women), not meeting laboratory eligibility criteria (8%, 203 women), and not meeting other clinical eligibility criteria (12%, 295 women). Only 58 (2%) of women were ineligible based on a grade 2 or higher pelvic examination finding. Finally, site investigators exercised discretion to exclude potential subjects for whom study participation would be unsafe or whose participation would complicate interpretation of study outcome data or otherwise interfere with achieving the study objectives. This discretion was exercised for 753 women (31% of those screened and not enrolled). Most often, this discretion was used to enroll women committed to the study objectives and its intensive, longitudinal follow-up schedule.

**Table 3:** Summary of subjects screened.

	Malawi	South Africa	Uganda	Zimbabwe	All Countries
**Participants Screened**	399	3471	408	1238	5516
Participants enrolled	272 (68%)	1426 (41%)	253 (62%)	678 (55%)	2629 (48%)
Participants not enrolled	127 (32%)	2045 (59%)	154 (38%)	560 (45%)	2887 (52%)
Participant did not complete all screening procedures	0 (0%)	279 (8%)	31 (8%)	68 (6%)	378 (7%)
Participant is eligible but declined enrollment	1 (<1%)	35 (1%)	4 (1%)	15 (1%)	55 (1%)
Participant not eligible	126 (32%)	1731 (50%)	120 (29%)	477 (39%)	2454 (44%)
Participant < 18 or > 45 years old	0 (0%)	0 (0%)	0 (0%)	0 (0%)	0 (0%)
Plans for relocation/travel	8 (6%)	37 (2%)	5 (4%)	18 (4%)	68 (3%)
Participant is pregnant or planning to become pregnant	9 (7%)	108 (6%)	18 (15%)	68 (14%)	203 (8%)
Participant is breastfeeding	5 (4%)	22 (1%)	2 (2%)	2 (<1%)	31 (1%)
Participant has not had vaginal sex in the last 3 months	5 (4%)	41 (2%)	3 (3%)	7 (1%)	56 (2%)
Participant has enrolled in another research study in the last 60 days	0 (0%)	0 (0%)	0 (0%)	0 (0%)	0 (0%)
Participant has participated in VOICE or other HIV prevention trial in the past 12 months	0 (0%)	0 (0%)	0 (0%)	0 (0%)	0 (0%)
PEP exposure in the last 6 months	2 (2%)	4 (<1%)	0 (0%)	0 (0%)	6 (<1%)
Participant is HIV-positive	19 (15%)	590 (34%)	27 (23%)	218 (46%)	854 (35%)
Participant declines effective method of contraception	5 (4%)	15 (1%)	6 (5%)	1 (<1%)	27 (1%)
Participant has a grade 2 or higher pelvic exam finding	3 (2%)	43 (2%)	2 (2%)	10 (2%)	58 (2%)
Participant does not meet laboratory eligibility criteria	8 (6%)	159 (9%)	9 (8%)	27 (6%)	203 (8%)
Participant does not meet other clinical eligibility criteria	6 (5%)	225 (13%)	6 (5%)	58 (12%)	295 (12%)
Other reason, including investigator decision	60 (48%)	553 (32%)	51 (43%)	89 (19%)	753 (31%)

## Discussion

African HIV-1 seronegative women at risk of HIV-1 acquisition from the general population were successfully enrolled into this phase III trial of the dapivirine vaginal ring for HIV-1 prevention. Participants were sexually active, with an important minority reporting >1 partner during the prior 3 months, and curable STI prevalence was high. The accrual of women for this study demonstrates the feasibility of identifying and recruiting women to determine the effectiveness and safety of the dapivirine vaginal ring as PrEP, and indicates that HIV-1 seronegative women are readily identifiable in peri-urban and urban settings in sub-Saharan Africa for targeted implementation and delivery of this microbicide, if demonstrated to be efficacious and safe for HIV-1 prevention.

CAPRISA 004, a study of pericoital vaginal use of a 1% tenofovir gel, demonstrated the efficacy of vaginal tenofovir gel for the prevention of HIV-1 acquisition by women in South Africa, providing the first proof-of-concept for the use of a topical microbicide [4]. This finding was followed closely by evidence of the efficacy of oral tenofovir-based PrEP in men who have sex with men and heterosexual men and women [4–8]. In these trials, HIV-1 protection was strongly correlated with PrEP adherence. In two additional trials of tenofovir-based PrEP, evaluating gel and oral formulations, efficacy was not seen and testing of participant blood samples revealed only a minority (<30%) were using the study products consistently [9,11,22]. These findings underscore the importance of adherence to oral and topical PrEP and for evaluating new PrEP strategies; the continuous dosing regimen provided by the dapivirine ring may address at least in part- address PrEP adherence challenges. Notably, in the ASPIRE population, approximately 40% of participants were less than 25 years of age at the time of enrollment, more than half were unmarried (59%), and over 40% reported recent sex unprotected by condoms. In the VOICE trial of HIV-1 seronegative heterosexual women, these baseline characteristics predicted higher HIV-1 incidence as well as lower product adherence [22].

In prior PrEP studies, multiple methods were used to assess adherence, including self-report, counts of returned unused pills or gel applicators, and audio computer-assisted self-interview. Nonetheless, in several of these studies, fewer than half of all participants disclosed non-adherence or barriers to use, and products were returned in a manner consistent with high product use [22]. These findings support the need for measures of product adherence that do not rely solely on self-report or are easily manipulated by participants. In the ASPIRE study, plasma samples and returned used rings are collected for dapivirine testing and will provide an objective marker of product adherence.

All participants in ASPIRE receive a comprehensive package of HIV-1 risk reduction services, including condoms, counselling about risk reduction, STI diagnosis and treatment, offers for partner testing, and referrals for further care throughout study follow-up. Although self-reported condom use for last vaginal sex act at baseline was high in our cohort (57%), women may have over-reported condom use as a result of social desirability bias. Although condoms have proven efficacy, their effectiveness is limited due to inconsistent and incorrect use [23]. Moreover, condom use may be outside of the locus of female control, antithetical to constructs of trust and love and mediated by transactional exchanges of gifts or cash [24]. As female-initiated methods, microbicides are positioned to reduce women’s vulnerability to HIV-1 infection by empowering women to control their use [25,26]. High HIV-1 prevalence at screening across the study countries underscores the urgency around identification of interventions for women to protect themselves from HIV-1 acquisition.

One of the defining characteristics of microbicides has been its potential to be used clandestinely, or without the explicit acquiescence of a male partner. Interestingly, however, the majority of women in ASPIRE report telling their male partners about their planned study participation and anticipated ring use. Additional work during the trial will explore disclosure of ring use, male partners’ perceived attitudes and reactions, and the influence of male partners on women’s adherence to ring use.

Many factors, aside from susceptibility to HIV-1 infection, motivate young women to enroll into HIV-1 prevention clinical trials. Perceived access to quality health services including HIV-1 counselling and testing, health education, transportation reimbursement, peer pressure and altruism are all cited as motivators for study participation [27]. Given the long duration of HIV-1 prevention trials, participants’ life circumstances often change over time and consideration for management of commitment to visit schedules may be explored in advance of enrollment in the context of life plans (e.g. potential future employment, education or marriage). Careful assessment of all women presenting for enrollment by site investigators during the screening process was encouraged and executed across ASPIRE sites in an attempt to recruit individuals committed for the duration of the study.

Approximately 20% of women who screened out were due to clinical and laboratory related eligibility criteria. If dapivirine-based PrEP is shown to be safe and efficacious in ongoing trials, its safety will subsequently need to be assessed in HIV-1 susceptible persons who are less optimally healthy than those selected for this trial, as well as pregnant and lactating women. Bridging studies are already being planned for lactating women to respond to these questions.

Participants in prevention research on stigmatized diseases such as HIV/AIDS face social and psychological risks, especially marginalized and vulnerable populations [28,29]. However, our results demonstrate that a cohort of 2629 African heterosexual women at risk of HIV-1 transmission was successfully recruited into a placebo-controlled safety and effectiveness trial of the dapivirine vaginal ring. If this microbicide is demonstrated to be safe and effective in ASPIRE and a second ongoing phase III trial (The Ring Study), implementation of this intervention could be targeted to at-risk women in an effort to curb the HIV epidemic. For persons at ongoing risk of HIV-1 infection, topical and oral antiretroviral prophylaxis provides a time-limited HIV-1 prevention strategy under their control. As with all prevention strategies, PrEP is only effective if used, and maximum PrEP benefits, at both individual and population levels, will likely be achieved by combining PrEP with other effective HIV-1 prevention interventions. Long-acting microbicide-based PrEP products, if found to be well tolerated and effective, could simplify dosing regimens, thereby reducing user- dependent adherence challenges. A monthly vaginal ring may offer women a novel prevention strategy that will allow for effective prevention of HIV-1 acquisition.
